# Editorial: Artificial intelligence in rheumatology and musculoskeletal diseases

**DOI:** 10.3389/fmed.2024.1402871

**Published:** 2024-04-05

**Authors:** Edoardo Cipolletta, Maria Chiara Fiorentino, Florentin Ananu Vreju, Sara Moccia, Emilio Filippucci

**Affiliations:** ^1^Rheumatology Unit, Department of Clinical and Molecular Sciences, Polytechnic University of Marche, Ancona, Italy; ^2^Academic Rheumatology, University of Nottingham, Nottingham, United Kingdom; ^3^Department of Information Engineering, Polytechnic University of Marche, Ancona, Italy; ^4^Department of Rheumatology, University of Medicine and Pharmacy of Craiova, Craiova, Romania; ^5^Department of Excellence in Robotics and AI, The Biorobotics Institute, Scuola Superiore Sant'anna, Pisa, Italy

**Keywords:** artificial intelligence, convolutional neural network (CNN), imaging, rheumatology, arthritis, magnetic resonance imaging, ultrasound

The field of artificial intelligence (AI) in medicine has evolved dramatically over the past decade, paving the way for ground-breaking innovation. Recently, the potentiality of AI in rheumatology and musculoskeletal diseases has drawn the attention of the research community. In this Research Topic, various advancements, and applications of AI in the field are showcased, shedding light on this relatively unexplored domain.

Traditionally, the role of a physician in diagnosing and treating health issues has relied heavily on human intelligence, intuition, and experience. However, the process of formulating a diagnosis, the crucial first step in medical intervention, often proves to be challenging and subjective. It involves gathering information through patient history, physical examination, laboratory tests, and imaging studies, followed by the interpretation of this data. Herein lies the opportunity for AI to augment human intelligence in solving health-related problems by managing big data in medicine to support clinicians in the actual clinical practice.

AI could boost clinicians' knowledge and support their decision-making to create an "artificially enhanced rheumatologist.” AI algorithms, particularly data-driven ones such as machine and deep learning, leverage vast amounts of patient data to predict outcomes and facilitate informed decision-making. These algorithms can analyse physician notes (1), laboratory results (2), and imaging studies (3, 4) to aid in the management of rheumatic diseases (5) and to predict patients' prognosis (6). For instance, rule-based natural language processing and text extraction from large datasets of unstructured information have provided insights into treatment patterns and outcomes for rheumatic diseases. Motta et al. have shown that patients with rheumatoid arthritis (RA), psoriatic arthritis (PsA), and psoriasis treated with a multidisciplinary approach are more likely to receive innovative treatments or glucocorticoids, possibly reflecting more complex cases.

Machine-learning algorithms show promise for processing advanced imaging techniques, such as fluorescence optical imaging (FOI), which enables the visualization and analysis of inflammation in rheumatic diseases. In the paper authored by Rothe et al., machine-learning algorithms were applied to reveal the importance ranking of imaging features in FOI and find the most informative findings for accurately diagnosing different rheumatic diseases. Furthermore, deep-learning models can segment relevant structures in ultrasound images and help in educating young sonographers (7) and in the acquisition and interpretation (8, 9) of ultrasound images. Overgaard et al. have developed an AI model which can identify osteophytes and score them using a validated semiquantitative system for ultrasound imaging. The agreement performance between an expert reader and the AI algorithm was found to be slightly higher than previously found agreements between experts when assessing osteophytes on ultrasound images of the hand joints, with potential applications in the diagnosis and monitoring of hand osteoarthritis.

Besides, AI can assist clinicians during remote consultations and telemedicine. Phatak et al. have evaluated the performance of a convolutional neural network on standardized smartphone photographs in detecting inflammation in three hand joints (i.e., wrist, PIP2 and PIP3) of patients with various rheumatic diseases and compared it to a rheumatologist's diagnosis. The good-to-excellent diagnostic accuracy of AI suggests a potential use of computer vision in screening and follow-up of inflammatory arthritis involving the hands.

Additionally, the collection contains three insightful reviews on the integration of AI in rheumatology. The first review authored by Nicoara et al. emphasizes AI's role in the early diagnosis of conditions like RA and axial spondyloarthritis through magnetic resonance imaging. The second review by Gilvaz and Reginato highlights AI's potential in leveraging patient data for optimized management strategies in RA from diagnosis to treatment, while the third review by Dinescu et al. explores AI's applications in musculoskeletal ultrasonography, offering automated solutions for disease monitoring. Together, these reviews underscore the transformative impact of AI in various facets of rheumatology, from diagnosis to treatment optimization and imaging standardization.

The use of AI in rheumatology offers several advantages, including increased efficiency, reduced variability, and improved accessibility to healthcare services. It has the potential to streamline medical care, assist clinicians in decision-making processes, and enhance patient outcomes. However, challenges such as technical limitations, regulatory hurdles, small study size, and risk of overfitting must be addressed before AI can be integrated into routine clinical practice.

In conclusion, while the full potential of AI in rheumatology has yet to be unlocked, ongoing advancements and successful pilot studies indicate a promising future for AI-driven patient care. With continued research and collaboration, AI has the potential to revolutionize the diagnosis, treatment, and management of rheumatic and musculoskeletal diseases, ultimately improving the lives of patients worldwide.

## Author contributions

EC: Conceptualization, Writing – original draft, Writing – review & editing. MF: Writing – original draft, Writing – review & editing. FV: Writing – original draft, Writing – review & editing. SM: Writing – original draft, Writing – review & editing. EF: Writing – original draft, Writing – review & editing.
